# Prevalence of Risk Factors for Suicide Among Veterinarians — United States, 2014

**Published:** 2015-02-13

**Authors:** Randall J. Nett, Tracy K. Witte, Stacy M. Holzbauer, Brigid L. Elchos, Enzo R. Campagnolo, Karl J. Musgrave, Kris K. Carter, Katie M. Kurkjian, Cole Vanicek, Daniel R. O’Leary, Kerry R. Pride, Renee H. Funk

**Affiliations:** 1 Division of State and Local Readiness, Office of Public Health Preparedness and Response, CDC; 2 Montana Department of Public Health and Human Services; 3 Department of Psychology, Auburn University; 4 Minnesota Department of Health; 5 Mississippi Board of Animal Health; 6 Wyoming Department of Health; 7 Pennyslvania Department of Health; 8 Idaho Department of Health and Welfare; 9 Virginia Department of Health; 10 Nebraska Department of Health and Human Services; 11 Emergency Preparedness and Response Office, National Institute of Occupational Safety and Health, CDC

Veterinarians are believed to be at increased risk for suicide compared with the general population (1). Few data on the occurrence of suicidal behavior and suicide risk factors among U.S. veterinarians are available. Veterinarians participating in two wellness summits held during September 2013 concluded that more research is needed on veterinarians and their mental health (2).

During July 1–October 20, 2014, an anonymous, Web-based questionnaire was made available through the Veterinary Information Network (VIN), an online community for veterinarians; VIN News Service; *JAVMA News*; and monthly e-mail messages to veterinarians in 49 states (Maine was excluded) and Puerto Rico sent through the state’s veterinary medical association, agriculture or livestock department, or health department. The questionnaire asked respondents about their experiences with depression and suicidal behavior, and included standardized questions from the Kessler-6 psychological distress scale that assesses for the presence of serious mental illness (3). Respondents with nonresponses were included in the denominators when calculating prevalence estimates.

Responses were received from 10,254 currently employed veterinarians (10.3% of all employed U.S. veterinarians). The most commonly reported age category was 30–39 years (28.8%), and 31.3% were male. Thirty-four percent reported practicing veterinary medicine for <10 years, 24.6% for 10–19 years, 21.6% for 20–29 years, and 19.8% for ≥30 years. Most (68.6%) respondents practiced small animal medicine, and 37.8% were practice owners. In comparison, 44.4% of U.S. veterinarians are male, and 66.6% practice small animal medicine exclusively (4).

Approximately 6.8% (95% confidence interval [CI] = 5.9%–7.7%) of male and 10.9% (CI = 10.2%–11.6%) of female respondents were characterized as having serious psychological distress based on the Kessler-6 psychological distress scale, compared with 3.5% of male and 4.4% of female U.S. adults, respectively (5). Since graduating from veterinary school, 24.5% and 36.7% (CIs = 23.0%–26.0%, 35.6%–37.8%) of male and female respondents reported experiencing depressive episodes, respectively, 14.4% and 19.1% (CIs = 13.2%–15.7%, 18.2%–20.0%) suicidal ideation, and 1.1% and 1.4% (CIs = 0.7%–1.5%, 1.2%–1.7%) suicide attempts. In comparison, male and female U.S. adults had a lower lifetime prevalence of depressive episodes (15.1% and 22.9%, respectively) and suicidal ideation (5.1% and 7.1%) but a higher prevalence of suicide attempts (1.6% and 3.0%) (6,7).

The findings in this report are subject to at least two limitations. First, the small number of veterinarians who responded compared with the number of those potentially eligible increases the likelihood of nonresponse bias. Second, the possibility exists for social desirability bias. Both of these factors could lead to overestimation or underestimation of the actual prevalence of risk factors for suicide among U.S. veterinarians. Nevertheless, these data suggest that nearly one in 10 U.S. veterinarians might suffer from serious psychological distress and more than one in six might have experienced suicidal ideation since graduation. Additional data, particularly data from representative samples, are needed to further characterize the underlying risk factors for suicidal behavior among veterinarians and identify effective prevention methods.
